# Autophagy: Are Amino Acid Signals Dependent on the mTORC1 Pathway or Independent?

**DOI:** 10.3390/cimb46080519

**Published:** 2024-08-13

**Authors:** Chenglong Jin, Min Zhu, Jinling Ye, Zhiwen Song, Chuntian Zheng, Wei Chen

**Affiliations:** 1 State Key Laboratory of Swine and Poultry Breeding Industry, Guangzhou 510640, China; jinchenglong1992@163.com (C.J.); yejl2014@163.com (J.Y.); zhengchuntian@gdaas.cn (C.Z.); 2 Institute of Animal Science, Guangdong Academy of Agricultural Sciences, Guangzhou 510640, China; 3 Key Laboratory of Animal Nutrition and Feed Science in South China, Ministry of Agriculture and Rural Affairs, Guangzhou 510640, China; 4 Guangdong Provincial Key Laboratory of Animal Breeding and Nutrition, Guangzhou 510640, China; 5 Key Laboratory of Animal Genetics, Breeding and Reproduction in the Plateau Mountainous Region, Ministry of Education, College of Animal Science, Guizhou University, Guiyang 550025, China; mzhudky@gzu.edu.cn (M.Z.); zws2020gzu@163.com (Z.S.)

**Keywords:** amino acid, autophagy, molecular mechanism, mTORC1

## Abstract

Autophagy is a kind of “self-eating” phenomenon that is ubiquitous in eukaryotic cells. It mainly manifests in the damaged proteins or organelles in the cell being wrapped and transported by the autophagosome to the lysosome for degradation. Many factors cause autophagy in cells, and the mechanism of nutrient-deficiency-induced autophagy has been a research focus. It has been reported that amino-acid-deficiency-induced cellular autophagy is mainly mediated through the mammalian rapamycin target protein complex 1 (mTORC1) signaling pathway. In addition, some researchers also found that non-mTORC1 signaling pathways also regulate autophagy, and the mechanism of autophagy occurrence induced by the deficiency of different amino acids is not precisely the same. Therefore, this review aims to summarize the process of various amino acids regulating cell autophagy and provide a narrative review on the molecular mechanism of amino acids regulating autophagy.

## 1. Introduction

Amino acids, as the basic unit of protein synthesis, are necessary for the metabolism of the growth and development of the body [1,2,3]. Once amino acids are deficient, autophagy and other adverse reactions will be induced [4,5,6]. However, the relationship between autophagy and the deficiency or supply of different amino acids is multifaceted and it is uncertain if it affects the mammalian target of rapamycin complex 1 (mTORC1) pathway regulation [5,7,8].

Autophagy is characterized by damage in the cytoplasm and mitochondria, endoplasmic reticulum, peroxisome, and other organelles, as well as by the surrounding of intracellular pathogens by autophagosomes and their degradation by lysosomes [9]. It has been long considered as an essential protein degradation pathway. Similarly, the ubiquitin proteasome system (UPS) is another important factor in intracellular protein degradation. Still, the difference between the two is that UPS targets a single short-lived protein, and autophagy as a whole process degrades many long-lived proteins and organelles in cells [10,11]. Autophagy is a crucial mechanism for protein homeostasis and quality control.

Previous studies have reported that autophagy is stimulated by various pathological and physiological stresses, especially undernourishment [12,13]. Starvation-induced autophagy is an evolutionary conservative response of eukaryotes, which degrades proteins, carbohydrates, and lipids, makes cells adapt to their metabolism, and meets energy requirements [5,9]. Furthermore, amino-acid- or other nutrient-deficiency-induced autophagy effectively protects cells by blocking mitochondrial-induced apoptosis [4,5,7,8,9,10,13,14,15,16]. As a dynamic circulation system, autophagy provides substrates and energy for producing new proteins and membranes to promote the survival of cells under starvation conditions [17,18].

## 2. Autophagy

### 2.1. Classification of Autophagy

**Figure 1:**
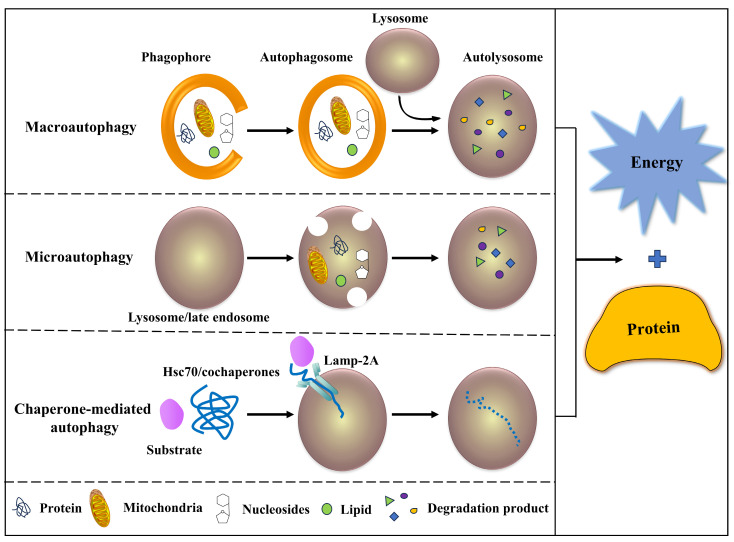
Different categories of autophagy. Macro-autophagy: proteins, lipids, and organelles such as mitochondria, etc., were surrounded by phagophores (also known as isolation membranes) to build an autophagosome, subsequently fused with lysosomes to form an autolysosome, the internal nutrients, and organelles were accordingly degraded. Micro-autophagy: proteins, lipids, and organelles were swallowed by invagination of the lysosome and late endosome. Chaperone-mediated autophagy (CMA): Cytosolic Hsc70 and cochaperones identified the substrate protein (including a KFERQ-like pentapeptide sequence), then translocated into the lysosomal lumen with lysosomal Lamp-2A. All degradation products were used for protein synthesis, energy production, etc.

Autophagy can be classified into the following three categories (Figure 1) based on the pathways by which degraded substrates are transported into the lysosome: (1) Macro-autophagy: the transfer of soluble cytoplasm and damaged organelles to lysosomes by using intermediate organelles called autophagy corpuscles, which are products of membranes [13,14]. Autophagy and lysosomes fuse to form autophagy lysosomes and degrade the substances in them. (2) Micro-autophagy: the lysosome’s membrane directly encapsulates long-lived proteins and degrades in the lysosome [9,10]. (3) Chaperone-mediated autophagy (CMA): the lysosome directly engulfs small pieces of cytoplasm through the concave lysosomal membrane [19]. In CMA, the substrate protein containing the KFERQ-like pentapeptide sequence first binds to the cytoplasmic Hsc70 and then translocates to the lysosomal lumen, which is recognized by a lysosomal receptor, the lysosomal-related membrane protein 2A, leading to its unfolding and degradation [20]. The substrate of CMA is a soluble protein molecule, which has selectivity in protein removal, while the former two have no apparent selectivity [21,22]. The three degradation products of autophagy can be used in new protein synthesis, energy production, or gluconeogenesis. Among these, macro-autophagy is the most important form and the most widely studied [4,5,7,8,9,10,13,14,15,16,17,18,19].

### 2.2. Characteristics of Autophagy

(1) Autophagy is a part of the digestion of cells, which seems terrible for cells at first sight. In fact, under normal circumstances, autophagy is performed chiefly at a basal rate unless there are some predisposing factors [23,24,25]. These factors, such as low nutrient content, hypoxia, and cytokine concentration, may be extracellular. There are also some intracellular ones, such as metabolic stress, aging or broken organelles, misfolding, or accumulation of proteins [4,5,7,8,9]. Because these factors often exist, cells maintain a low and fundamental autophagy to maintain stability. (2) The process of autophagy is very rapid. The formation of autophagosomes can be observed after 8 min of induction, and autophagy lysosomes are degraded and disappear after 2 h, which is conducive to the rapid adaptation of cells to harsh environments [10,11,13]. (3) Inducible characteristics: these are manifested in two aspects. One is the formation of autophagy-related proteins, which is the preparation stage. The second is the rapid and massive formation of autophagosomes, which is the execution stage [14,15,16,17,18,20]. (4) Batch degradation: batch degradation of autophagosomes is another significant difference from the UPS [10,11]. (5) The specificity of the “capture” cytoplasmic component: because the speed of autophagy is large and the amount is large, specificity is not the first consideration, which is compatible with the emergency characteristics of autophagy [13,14,23,24]. (6) Conservation: autophagy is conducive to the survival of cells, so it is generally preserved regardless of species or between various cell types (including tumor cells) [26].

### 2.3. Process of Autophagy

Autophagy is a process of phagocytosis of cytoplasmic proteins or organelles and their inclusion into vesicles and fusion with lysosomes to form autolysosomes, which degrade the contents of their inclusion to achieve the metabolic needs of cells themselves and the renewal of some organelles [9,10,11,13,14]. The specific process could be divided into four steps: (1) after the cell receives the autophagy signal, a membrane structure similar to a “liposome” will be formed somewhere in the cytoplasm, and then it will continue to expand. It is not spherical but flat, like a bowl composed of two layers of phospholipid bimolecular layers, which can be observed under an electron microscope, called a “phagophore”. (2) The phagophore extends, wrapping up proteins and organelles in the cytoplasm to form a closed autophagy [19]. (3) After autophagosomes are formed, they can fuse with phagocytic vesicles, pinosomes, and endosomes, but this step is not inevitable. (4) The autophagosome and lysosome are fused to form an autolysosome [17,19,23]. During this period, the inner membrane of the autophagy will be degraded by hydrolase in the lysosome, and the inner membrane of both the autophagy and lysosome will be integrated. The “goods” in autophagy will also be degraded, and the degradation products (amino acids, fatty acids, etc.) will be transported to the cytoplasm for cell reuse [23,24,26]. Some of the residues will be excluded from the cell, while others will be retained in the cell [27,28,29,30].

### 2.4. Regulation of Autophagy

Yeast is a simple eukaryotic cell, which makes it relatively easy to manipulate genes. As a result, many studies on autophagy used yeast as a model. In these studies, it has been found that the autophagy-related gene (Atg) and its corresponding protein play an essential role in different stages of autophagy [4,8,9,19,23].

Moreover, the mammalian target of rapamycin (mTOR), as a sensor of energy and nutritional status, is a typical serine/threonine kinase [31,32]. Under nutrient-rich conditions, mTOR complex 1 (mTORC1) binds to Atg1/Unc-51, like autophagy, activating kinase 1 (ULK1, the Atg1 named in mammalian), which directly inhibits downstream signals of autophagy [4,8]. Meanwhile, mTORC1 allows Atg13 to achieve phosphorylation, inhibiting downstream signals of autophagy. Because the affinity of highly phosphorylated Atg13 and Atg1 will be weakened, thereby reducing Atg1 kinase activity, downstream autophagy signals can be suppressed [33]. However, under starvation, mTORC1 inhibition leads to Atg13 dephosphorylation and close binding with Atg1. The close binding of Atg13 and Atg1 will enhance the activity of Atg1 kinase and activate the down-stream signal of autophagy [34]. In addition, ULK1 induces its phosphorylation by binding to raptor [35], thereby inhibiting the kinase activity of mTORC1.

**Figure 2:**
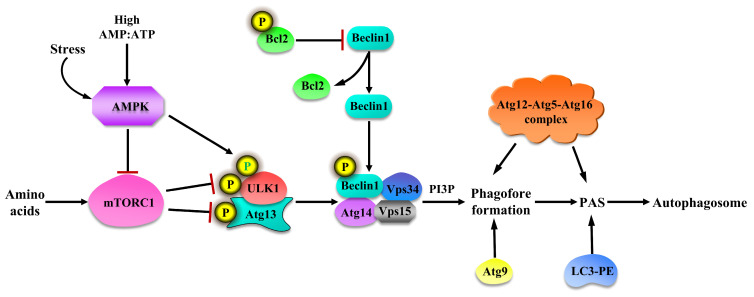
Regulation of autophagy in response to amino acids. High AMP and stress activate AMPK and depresses mTORC1, and AMPK can directly promote autophagy by phosphorylation of ULK1. Whereas rich amino acids could stimulate mTORC1 activation and suppress autophagy, mTORC1 blocks the occurrence of autophagy by phosphorylation of ULK1 and Atg13. Bcl2 is an inhibitor of autophagy. The interaction between Bcl2 and Beclin1 is broken by Bcl2 phosphorylation. Beclin1 is released and moved to the Vps34-Vps15 complex. Phosphorylation Be-clin1 activates Vps34 kinase activity and production of PI3P and contributes to nascent autophagy. Additionally, phagophore formation needs Atg9 and Atg12-Atg5-Atg1 complex, and LC3-PE also participates in pre-autophagosomal structure (PAS) construction and further forms autophagosomes.

Adenosine monophosphate-activated protein kinase (AMPK) mediates upstream signals of mTORC1, and mTORC1 controls the cell-energy-sensing pathway [36]. At a high concentration of AMP and its effect on energy consumption, AMPK is activated, which inhibits mTORC1 and promoted autophagy [37]. The activation of autophagy promotes the activation of the ULK1 complex and phosphorylates Beclin1 on Ser14, thereby enhancing the activity of Beclin1-Atg14-Vps34-Vps15 class III PI3K core complexes and promoting the nucleation of autophagosomes [38]. In addition, Bcl-2 binds to the BH3 domain of Beclin1 to inhibit autophagy under the condition of rich nutrition. The phosphorylation of Bcl-2 and Beclin1 destroys this interaction and releases Beclin1 [39]. When nutrient-rich, Atg9 shuttles between Golgi and endosomes, whereas when nutrient insufficient, Atg9 is distributed in the nuclear compartment, providing a membrane source for the formation of autophagosomes [40,41]. The two ubiquitin-like protein systems Atg12-Atg5-Atg16 (localized on the outer membrane of autophagy) and microtubule-associated protein 2 light chain 3 (LC3-II, named Atg8 in yeast and its mammalian homologous protein named LC3)-phosphatidylethanolamine (PE) are involved in the autophagy extension phase [42,43]. Under the catalysis of Atg7 and Atg10 enzymes, Atg12 and Atg5 are tightly bound through isopeptide bonds to form the Atg12-Atg5 complex. Through non-covalent bonding, Atg5 further binds to the spiral-helix region of Atg16 to form the Atg12-Atg5-Atg16 complex [44,45]. Atg16 (containing a coiled–coiled structure) undergoes homo-oligomerization through its unique structure to form a larger complex, which then binds to the pre-autophagosomal structure (PAS) and participates in the extension of the PAS [46]. Through the interaction of Atg7 and Atg3, Atg8/LC3 is covalently linked with PE to form the complex LC3-II-PE, which then binds to the membrane and participates in the extension of PAS [47]. The synthesis and processing of LC3 are often used to monitor the progress of autophagy because LC3-II is in mature autophagy until it merges with lysosome to produce autophagy. The regulation of autophagy is shown in Figure 2.

## 3. Autophagy: Amino Acid Signals Dependent on mTORC1 Pathway

Amino acids play an important role in the metabolism of nutrients and the energy supply for the body through the biological activity of themselves or their metabolites, ultimately regulating the growth and development of animals [48]. If the supplementation of amino acids is deficient, autophagy would be the most characteristic phenotype [3,4,5]. However, the specific mechanisms of autophagy caused by different amino acid deficiencies are not the same. At present, more studies are based on lysine (Lys), leucine (Leu), arginine (Arg), and glutamine (Gln).

mTORC1 is an evolutionarily conserved serine/threonine kinase indispensable in controlling protein metabolism, cell growth, and autophagy [49,50]. Moreover, it negatively regulates autophagy through the phosphorylation of ULK1 and Atg13 [51]. Under nutrient-rich conditions, mTORC1 supports cell growth and inhibits autophagy. In contrast, mTORC1 is inhibited when the cells are under nutrition starvation and induces autophagy to provide energy for its metabolism [52].

As early as 1977, Mortimore et al. found that amino acid deficiency increased autophagy in rat liver [53]. Branched chain amino acids (BCAAs), especially Leu, have an inhibitory effect on the occurrence of autophagy [54]. The mechanism of amino acids to inhibit autophagy is to increase mTORC1 activity in two ways. One way is that amino acids are linked to lysosomes by a complex called the ragulator, which acts as a guanine nucleotide exchange factor and activates the rag triphosphate guanosine (GTP) enzyme in Ras-related small GTPases (Rag) A when it is sufficient [32]. Rag family proteins are members of the Ras family of GTP enzymes. They form heterodimers from four members (RagA, RagB, RagC, and RagD). RagA and RagB bind to GTP, and RagC and RagD bind to guanosine diphosphate (GDP) [32,48]. The other way is that amino acid intake affects mitogen-activated protein kinase kinase kinase kinase 3 (MAP4K3) in Ste proteins. An exogenous increase in amino acid levels improves the activity of Rag GTPase and MAP4K3 [55]. The Rag GTPase complex promotes mTORC1 to connect with the lysosome and collects a large amount of Ras homologous protein (Rheb) rich in the brain to activate mTORC1 [56]. Rheb is negatively regulated by the tuberous sclerosis complex (TSC) 1/2, which is a GTPase-activating protein for Rheb [32,48,52,55,56]. mTORC1 leads to the dissociation of the ULK1 active complex. Unable to form LC3-Ⅱ, the autophagosome membrane fails to extend, autolysosome formation is blocked, and autophagy is inhibited. In this process, the transcription regulator ribosomal protein S6 kinase (S6K) is also activated to synthesize proteins [4].

### 3.1. Lys in Regulating Autophagy with mTORC1 Pathway

Lys is an essential component of protein synthesis and promotes the absorption and utilization of other nutrients [2,6,57]. It was found that the growth speed of piglets fed with a low-Lys diet was slower than that of piglets fed with a standard diet [31,57]. The muscle fiber density and muscle quality in the Lys deficiency group were also significantly reduced [58]. Lys deficiency reduces the growth of skeletal muscle, which may be due to insufficient Lys activating the autophagy–lysosomal pathway, resulting in the autophagy of skeletal muscle cells, which makes proteins degraded to resist the lack of intracellular amino acids caused by the external environment and other factors, and then to maintain intracellular amino acid balance in a short time [59]. According to Sato et al., Lys plays a role in preventing the breakdown of skeletal muscle protein [60]. In the absence of Lys, muscle protein decomposition and synthesis will speed up, leading to a decrease in muscle mass [61]. This even results in clinical symptoms such as muscle atrophy and myasthenia.

Studies have shown that the autophagy–lysosomal system is regulated by mTOR activity [62]. Sato et al. analyzed the phosphorylation levels of the mTORC1 downstream target proteins S6K1 and 4EBP1 in Lys-treated C2C12 myotubes [63]. After the C2C12 myotube was treated in a medium containing 10 mmol/L Lys for 30 min, the ratio of LC3-II to LC3-I was significantly reduced by 30% compared to that of the control group (the medium did not contain amino acids). The level of p-4EBP1 significantly increased, suggesting that Lys regulates the autophagy–lysosomal activity of the C2C12 myotube through the mTORC1-dependent pathway. When the supply of Lys was sufficient, Lys activated the mTORC1 signaling pathway to inhibit the activity of the autophagy–lysosomal system and reduced the degradation of myofibrillar protein in muscle. The synthesis of muscle protein is related to the activation of the mTORC1 pathway. Lang et al. proved that Lys supplementation improved the level of p-4EBP1, which indicates that Lys supplementation inhibits the activity of the autophagy–lysosomal system and promotes the synthesis of proteins by activating the mTORC1 signaling pathway in muscle cells [64]. It has been reported that Lys slowed down the degradation of myofibrillar protein in rats. After feeding 114 mg and 570 mg of Lys for every 100 g of body weight in fasted rats, it was found that the degradation rate of myofibrillar protein was significantly reduced, and the expression of LC3-II was also significantly reduced. However, the mechanisms of Lys inhibiting the degradation of myofibrillar protein are still unclear [59]. Please see Table 1.

### 3.2. Leu in Regulating Autophagy with mTORC1 Pathway

Leu is a neutral amino acid. As an essential branched chain amino acid, it cannot be synthesized in animals and can only be taken from food to meet the body’s need to maintain metabolic balance. Recent studies have shown that Leu not only regulates the metabolic level of proteins in mammals but also plays an essential role in maintaining the body’s immune and oxidative functions [65,66]. Many studies have linked protein breakdown caused by Leu deprivation with autophagy. Wu et al. reported that two members of the miR-17 microRNA family, miR-20a and miR-106b, may inhibit the expression of ULK1 in mouse myoblasts (C2C12) involved in the regulation of Leu-deprivation-induced autophagy [67]. Sato et al. found that Leu intake inhibited the activation of the autophagy–lysosomal system and prevented the degradation of myofibrillar protein [60]. Subsequent research also found that the ratio of LC3-Ⅱ to LC3-Ⅰ decreased significantly after Leu supplementation in C2C12 cells cultured in Dulbecco’s modified Eagle medium (DMEM) for 30 min, suggesting that Leu supplementation has a significant inhibitory effect on the autophagy of C2C12 cells. Amino acid deficiency causes autophagy in cells, and Leu is considered to be the most effective autophagy inhibitor [68].

Leu regulates autophagy mainly because it activates the mTORC1 signaling pathway, and then up-regulates its downstream target protein, thus inhibiting autophagy [69]. Recent studies have shown that the inhibition of Leu on autophagy depends on the mTORC1 signaling pathway, and its upstream target proteins, the Ras homologue enriched in the brain (Rheb) and the regulatory associated protein of mTOR (Raptor), are also necessary. Leu promotes the phosphorylation of mTORC1, and then phosphorylates its downstream target proteins 4EBP1 and S6K1 [70]. It also activates the target protein Rag upstream of mTORC1, so that inactive and diffusely distributed mTORC1 in the cell aggregated around its activating protein. Lang et al. and Yoshizawa et al. found that Leu supplementation enhances the mTORC1 activity of myocytes and then inhibits autophagy [64,71]. When C2C12 myotube cells were treated in Leu containing medium for 30 min, the level of p-4EBP1 could be significantly increased [63]. The microRNA microarray results of Wu et al. showed that 84 microRNAs were differentially expressed in C2C12 myotube cells after 4 h of Leu starvation. The prediction and analysis of these differentially expressed microRNA target genes show that the change in mTOR-autophagy signaling pathways induced by Leu deletion might be indirectly affected by a change in miR-20a and miR-106b [67]. Yan et al. found that the protein breakdown caused by Leu deprivation was related to autophagy. Barkor/Atg14 is a special autophagy binding protein. Under the condition of rich nutrition, its ability of binding to the autophagy membrane is inhibited, and after nutrition deprivation, the inhibition is removed. At the same time, rapamycin effectively reverses the inhibition of nutritional enrichment, suggesting that mTORC1 plays a vital role in the inhibition of autophagy [49]. Please see Table 1.

### 3.3. Arg in Regulating Autophagy with mTORC1 Pathway

Arg is a functional amino acid, which plays an important role in the body’s growth and development, physiological metabolism, and intestinal health [72]. Most melanoma cells require an exogenous supply of Arg for growth and proliferation. Arg deprivation inhibits mTOR signaling but leads to the activation of MEK and ERK. These changes are likely to lead to autophagy, a possible mechanism for survival through the intracellular arginine cycle [73]. Moreover, under conditions of heat stress, Arg induces autophagy with the decreased expression of p62 and p-mTOR/mTOR, and the increased expression of LC3B and Arg can reduce intestinal damage and protect intestinal integrity by promoting autophagy [74]. Additionally, Castejón-Vega et al. investigated the effects of autophagy and lysosomal integrity using skin fibroblasts obtained from Tay-Sachs and Sandhoff patients (GM2 gangliosidosis) with increased lysosome permeability. GM2 fibroblasts showed decreased mTOR signaling and decreased basal mTOR activity. Supplementing with Arg to provide positive nutritional signals could partially restore mTOR activity and alleviate cytopathological abnormalities [75]. These findings suggest that Arg regulates the autophagy process under mTOR modulation. Please see Table 1.

### 3.4. Gln in Regulating Autophagy with mTORC1 Pathway

Gln is a rich amino acid in the blood. Many tissues, especially skeletal muscle, synthesize Gln as an important carrier of carbon and nitrogen. In addition, it also plays a crucial role in maintaining the intestinal integrity of humans and animals [76,77]. Zhu et al. used pig intestinal epithelial cells to test the hypothesis that Gln deficiency induced autophagy and inhibited autophagy after Gln supplementation [78]. After cultured in normal medium for 2 days, the intestinal epithelial cells were transferred to a Gln-free medium, and the number of cells, the distribution of the autophagy, the expression of LC3, and the protein abundance of mTOR within 8 h were detected. In addition, the complement effect of 5 mmol/L Gln was evaluated. The results showed that Gln deficiency reduced the number of cells and increased the accumulation of autophagy and the expression of LC3-II within 8 h. These results support the view that Gln deficiency induces autophagy, interferes with amino acid metabolism in intestinal epithelial cells, and weakens the mTORC1 signaling pathway to inhibit protein synthesis and cell proliferation. Tan et al. found that during amino acid starvation, Gln needed to reactivate mTORC1 by autophagy, and Gln supplementation alone was sufficient to restore mTORC1 activity and lysosomal localization during long-term amino acid starvation [77]. The inhibition of Gln was eliminated by the treatment of autophagy inhibitors, suggesting that the activation of mTORC1 depended on autophagy. Please see Table 1.

**Table 1:** Amino-acid-regulated autophagy dependent on mTORC1 signaling pathway.

Amino Acids	Treatment	Samples	Description	References
Lys	10 mM Lys treated for 30 min	C2C12	Suppressed autophagy, activated mTORC1	[60,63]
	Rats fed with 10% casein diet supplemented by Lys	Rat muscle	Suppressed autophagy, activated mTORC1	[79]
Leu	10 mM Leu treated for 30 min	C2C12	Suppressed autophagy, activated mTORC1	[60,63]
	Fasted mice administered an oral gavage of Leu (1.35 g/kg BW)	Mouse muscle	Increased protein synthesis and mTORC1	[64]
	Fasted pigs were infused intra-arterially with Leu (0, 200, or 400 μmol/kg/h)	Pig muscle	Increased protein synthesis and mTORC1	[70]
	Oral administration of Leu in fasted rats	Rat muscle	Increased protein synthesis and mTORC1	[71,80]
Arg	Medium Arg deficiency	Melanoma cells	Induced autophagy, restricted mTORC1	[73]
	Diet Arg supplementation in heat-stress-treated rats	Rat intestine	Induced autophagy, restricted mTORC1	[74]
	Medium Arg supplementation in heat-stress-treated IEC-6	IEC-6	Induced autophagy, restricted mTORC1	[74]
	Arg-treated skin fibroblasts isolated from GM2 gangliosidosis	Skin fibroblasts	Recovered autophagy and mTORC1	[75]
Gln	Gln-treated MEFs and HepG2 cells under amino acid starvation	MEFs and HepG2 cells	Restored autophagy and mTORC1	[77]
	Gln deprivation treated IPEC-1	IPEC-1	Induced autophagy, restricted mTORC1	[78]

## 4. Autophagy: Amino Acid Signals Independent of mTORC1 Pathway

### 4.1. Lys in Regulating Autophagy without mTORC1 Pathway

Although the regulation of Lys on cell autophagy is mainly mediated through the mTORC1 signaling pathway, since the role of the mTORC1 pathway is limited, the lack of Lys to induce cell autophagy may also be mediated by signaling molecules other than mTOR [79]. It has been reported that protein kinase B (Akt) regulates protein degradation through the autophagy–lysosomal system [81]. Previous studies have also shown that Leu inhibited the activity of the autophagy–lysosomal system by up-regulating the level of Akt [82], but Sato’s research has fully confirmed that Lys is another amino acid that can activate the Akt signaling pathway [79]. Research by Zhao et al. showed that Akt, as a regulator, is indispensable in the process of autophagy in myotubes [83]. Therefore, Akt activation may play a key role in Lys regulating autophagy–lysosomal activity. Sato et al. treated C2C12 cells with Lys and found that the phosphorylation level of the Ser473 and Thr308 sites of Akt significantly increased [60]. When C2C12 cells were treated with Akt-specific inhibitors, Lys inhibited the degradation of myofibrillar protein by activating Akt.

Sato also evaluated the regulatory effect of Lys-induced Akt activation on autophagy–lysosomal activity by the ratio of LC3-II to total LC3. In C2C12 cells treated with Lys, the ratio of LC3-II to total LC-3 decreased, which could be eliminated after being treated with the Akt inhibitor, indicating that Lys inhibition of autophagy–lysosomal system activity depended on Akt activation [79]. Please see Table 2.

### 4.2. Leu in Regulating Autophagy without mTORC1 Pathway

Although the effect of Leu on autophagy is mainly realized by the mTORC1 signaling pathway, and its effect on protein metabolism and turnover is also dependent on other signaling pathways [80], there are few related studies. In order to determine whether the effect of Leu on autophagy of muscle cells will play a role through a signaling pathway other than mTORC1, Mordier et al. carried out relevant experiments. They treated myotube cells with Leu starvation for 3 h to detect the effect of rapamycin by incubating myotube cells with 50 nmol/L of rapamycin for 3 h. The effect of Leu starvation was examined, and it was found that there was a cumulative effect when two stimuli were applied simultaneously. In addition, the measurement of p-S6 ribosomal protein levels through an 8-h test found that its expression level was not affected by Leu deprivation, thereby further providing that Leu starvation induces muscle cell autophagy through a mTORC1-independent pathway. This indicates that Leu starvation may regulate autophagy through specific signaling pathways unrelated to mTORC1, and also regulate protein synthesis [84]. Please see Table 2.

**Table 2:** Amino-acid-regulated autophagy independent of mTORC1 signaling pathway.

Amino Acids	Treatment	Samples	Description	References
Lys	Oral administration of Lys (114 mg/100 g BW, sufficient) in fasted rats	Rat muscle	Suppressed autophagy, unchanged mTORC1	[59]
	10 mM Lys treated for 30 min	C2C12	Suppressed autophagy and AMPK	[60]
	SAMP8 fed with 3.0% Lys diet	SAMP8	Suppressed autophagy, unchanged mTORC1	[61]
Leu	10 mM-Leu-treated for 30 min	C2C12	Suppressed autophagy and AMPK	[60]
	MiR-20a- and miR-106b-treated Leu deficiency medium pre-cultured C2C12	C2C12	Suppressed autophagy, unchanged mTORC1	[67]
	Old rats fed a 15% protein diet supplemented with 4.5% leucine	Rat muscle	Suppressed autophagy, unchanged mTORC1	[82]
	Leu-deficiency-treated C2C12	C2C12	Induced autophagy, unchanged mTORC1	[84]
Arg	Medium Arg deficiency	Melanoma cells	Induced autophagy, activated AMPK	[73]
	IFN-γ treated BMECs	BMECs	Decreased Arg content, increased GCN2 pathway and autophagy	[85]
	Arg-deficiency-treated H4-II-E and Hep G2 cells	H4-II-E and Hep G2 cells	Induced autophagy, activated NO pathway	[86,87]
Gln	Atg5 or Atg7 knockdown HeLa cells with Gln supplementation	HeLa cells	Suppressed autophagy, unchanged mTORC1	[88]
	Gln-deficiency-treated porcine-circovirus-type-2-infected mice and PK 15 cells	Mice and PK 15 cells	Induced autophagy, activated JAK2/STAT3	[89]

### 4.3. Arg in Regulating Autophagy without mTORC1 Pathway

The discovery of Xia et al. proved for the first time that the depletion of Arg and the expression of general control nonderepressible 2 (GCN2) mediate interferonγ (IFN-γ)-induced autophagy, and the continuous activation of autophagy promotes the transformation of bovine mammary epithelial cells [85]. In addition, studies by Sarkar et al. and Angcajas et al. have confirmed that Arg regulates autophagy not only through the mTORC1 signaling pathway, but also through the nitric oxide (NO) signaling pathway [86,87]. NO is a cell signaling molecule commonly found in the cardiovascular, nervous, and immune systems and triggers a variety of physiological responses, such as blood flow regulation and tissue response to hypoxia. Arg is the only source of NO in the human body. In cells, Arg can be degraded into NO by nitric oxide synthase (NOS). Studies by Sarkar et al. have shown that NO reduces the activity of Jun N-terminal kinase 1 (JNK1) and the phosphorylation of B-cell lymphoma-2 (Bcl-2) and inhibits autophagy flux in mammalian cells in a manner dependent on IkB kinase β (IKKβ) and tuberous sclerosis complex 2 (TSC2) [86]. Rat liver cancer cells H4-II-E were starved in an Earl-balanced salt solution, and the ratio of LC3-II/LC3-I decreased by 35% after adding Arg. After treatment with Arg and rapamycin, it was found that the ratio of LC3-II/LC3-I decreased by 33%. There was no significant difference in autophagy inhibition in both cases, indicating that Arg may also inhibit autophagy through other signaling pathways, rather than relying on the mTORC1 signaling pathway [87]. Please see Table 2.

### 4.4. Gln in Regulating Autophagy without mTORC1 Pathway

Gln is associated with increased enlarged lysosome formation/dissipation by regulating TFEB nuclear/cytoplasmic translocation but has nothing to do with mTORC1 activity [88]. Gln deficiency induces autophagy, which is evident through increased LC3-II conversion and GFP-LC3 dot accumulation, and further triggers porcine circovirus type 2 (PCV2) infection. Subsequent studies have demonstrated that Gln deficiency activates autophagy via the activation of ROS-mediated JAK2/STAT3 signaling [89]. In addition, data in neurons show that in the presence of Gln synthetase1 (GS1), TOR signaling is reduced, even when Gln levels increase, so this may indicate that in terminally differentiated cells, GS1 induces a ‘starvation-like’ condition that facilitates autophagy without activating TORC1 [90]. Similarly, an earlier study showed that Gln regulated autophagy by growth factor signaling [91]. Therefore, Gln may also inhibit autophagy through other signaling pathways, rather than the mTORC1 pathway. Please see Table 2.

## 5. Defects in Studies on Amino-Acid-Regulated Autophagy

Many studies on autophagy have fully shown that a lack of nutrition, especially in amino acids, will lead to strong autophagy. However, it is unclear how individual amino acids, such as Lys, Leu, Arg, or Gln, respond to autophagy, which is also the point of investigating the relationship between amino acid nutrition and autophagy. It has been shown that Lys and Leu regulate protein synthesis through the mTORC1 signaling pathway, but Leu stimulates mTORC1 signaling significantly more than Lys [92], and the similarities and differences between Lys and Leu in the regulation of protein metabolism need further study to be clarified.

The effects of nutrients, especially amino acids, on autophagy have been confirmed by studies for many years. However, there are few studies on how amino acids enter the cell to alleviate autophagy and whether amino acids act as a signaling molecule or a nutrient substrate in the process of regulating autophagy. When intracellular nutrients are scarce, cells will initiate autophagy, degrade their own cellular components, and maintain the balance between anabolism and catabolism in a short period of time under special physiological conditions [93]. However, how cells sense the lack of amino acids and then regulate autophagy is still worthy of further study.

## 6. Summary and Prospects

Autophagy is a kind of dynamic cell process to remove long-lived proteins, damaged organelles, and cytoplasmic components. In recent years, autophagy has been a hot topic in the field of life science and clinical disease research. At present, the relationship between the degree of autophagy and the body’s advantages and disadvantages cannot be quantitatively analyzed. The induction of autophagy by amino acid deficiency is mainly achieved by inhibiting the mTORC1 signaling pathway. The study on the molecular mechanism of how mammalian cells perceive amino acid deficiency to promote the formation of autophagy bodies, form autophagy lysosomes, then start the whole process of autophagy, and how autophagy-related-regulators receive and transmit signals downstream, is fiercely in progress. An in-depth study on the effect of amino acids, especially essential amino acids on autophagy, will help us to understand the precise regulation of nutrition more comprehensively and provide a theoretical basis for animal husbandry and medical research.

## Data Availability

Not applicable.
